# Weber Texture Local Descriptor for Identification of Group-Housed Pigs

**DOI:** 10.3390/s20164649

**Published:** 2020-08-18

**Authors:** Weijia Huang, Weixing Zhu, Changhua Ma, Yizheng Guo

**Affiliations:** 1 School of Electrical and Information Engineering, Jiangsu University, Zhenjiang 212013, China; huangweijia@just.edu.cn (W.H.); mch982004@126.com (C.M.); 2 School of Electronics and Information, Jiangsu University of Science and Technology, Zhenjiang 212003, China; 3 Nanjing Normal University Taizhou College, Taizhou 225300, China; guoyizheng0523@163.com

**Keywords:** Weber texture local descriptor, group-housed pigs, pig identification

## Abstract

The individual identification of group-housed pigs plays an important role in breeding process management and individual behavior analysis. Recently, livestock identification methods based on the side view or face image have strict requirements on the position and posture of livestock, which poses a challenge for the application of the monitoring scene of group-housed pigs. To address the issue above, a Weber texture local descriptor (WTLD) is proposed for the identification of group-housed pigs by extracting the local features of back hair, skin texture, spots, and so on. By calculating the differential excitation and multi-directional information of pixels, the local structure features of the main direction are fused to enhance the description ability of features. The experimental results show that the proposed WTLD achieves higher recognition rates with a lower feature dimension. This method can identify pig individuals with different positions and postures in the pig house. Without limitations on pig movement, this method can facilitate the identification of individual pigs with greater convenience and universality.

## 1. Introduction

The identification of group-housed pigs plays an important role in breeding process management and individual behavior analysis. The spread of infectious diseases is a huge threat to livestock farming. In order to detect the early symptoms of swine disease in time, people need to conduct effective monitoring of the group-housed pigs suffering from disease [1,2] through methods such as temperature measurement by infrared ray, detection of cough [3,4], calculation of the amount of drinking water [5], eating and drinking behavior recognition [6,7], and behavioral change measurement [8]. In this process, one of the most important basic tasks is to distinguish different pigs and identify pigs with abnormal behavior.

Electronic ear tags based on radio frequency identification (RFID) technology are widely used in livestock identification [9,10]. Readers can identify a large number of tags quickly and efficiently. However, the ear tag is an invasive method. It’s costly and might be lost in some cases [11]. Moreover, since pigs live together in groups, it is hard to identify each pig accurately at the same time if more than one pig enters the card reader range.

The identification method based on machine vision overcomes the limitations of electronic ear tags. In the early work, pigs were marked on their backs and sides with different colored spray paints. Jover et al. [12] segmented the image in the red-green-blue (RGB) color space and then recognized the color patterns using another color space. The position of piglets in a farrowing pen was detected. Kashiha et al. [13] used the ellipse fitting method to locate pigs. The paint patterns on the pigs’ backs were used to identify different individuals. However, due to the dirty environment of the pigsty and the growth of pigs, these markers are difficult to preserve well for a long time and cannot be effectively applied in practice.

Recent years have seen development in livestock recognition technology based on biometrics and machine learning [14], such as muzzle print recognition [15], iris recognition [16], face recognition, and so on. Zhao et al. [17] collected side images of cows passing through a fixed narrow channel. The convolutional neural network (CNN) was used for cow recognition. Shen et al. [18] employed the you only look once (YOLO) model to detect the cow in the side-view image and fine-tuned a CNN model to classify each individual cow. Hansen et al. [19] collected face images of pigs and used the face recognition network of Fishface, visual geometry group (VGG), and CNN to identify pigs. Marsot et al. [20] automatically detected each pig’s face and eyes by a cascade classifier based on Haar features and a shallow convolution neural network; then, they identified pigs by deep convolution neural network. These methods based on biometrics are stable and non-invasive. However, there are restrictions on image collection. Livestock usually need to go to a specific location or take specific poses. It’s difficult to be applied to surveillance videos of group-housed pigs. Furthermore, the training of deep convolution network needs massive data and great computational expense. When a new individual is added to the pig farm, image collection and training should be done again, which is time-consuming and inconvenient.

To solve the above problems, top view monitoring videos of group-housed pigs are collected, and a model based on the pigsty is established. Pigs move freely in the pigsty and the local texture features formed by the hair, skin lines, and spots on the body surface of pigs are used for the identification of group-housed pigs. Due to the uneven illumination and complex background in the actual pig farm, the accuracy of color and shape feature extraction is easy to be affected. Therefore, in this paper, the texture feature—which does not depend on the change of color or brightness—is extracted and pig identification based on appearance features is studied. The recognition method based on appearance features has low computational complexity and does not need training. Moreover, it is invariant to texture scale, translation and rotation, and illumination change [21]. Research on more powerful local feature descriptors has always been a hotspot of the appearance feature method [22]. In our previous work, the pig identification method based on Gabor and local binary pattern (LBP) was proposed [23]. However, the multi-scale feature of Gabor has a higher feature dimension and needs a large amount of computation. In this paper, a Weber texture local descriptor (WTLD) is proposed to enhance the feature description capability by calculating the differential excitation and multi-directional information of the pixel and fusing the local structure feature of the main direction. An adaptive threshold is used to quantify and encode, and a dense descriptor is constructed, which has higher recognition results and lower feature dimension.

## 2. Materials and Methods

**Figure 1:**
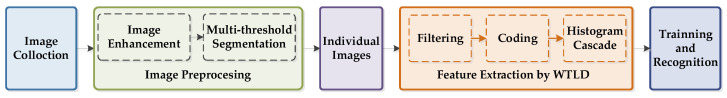
The flow diagram of the proposed method.

This paper proposes a novel method of group-housed pigs recognition based on WTLD. The framework is illustrated in Figure 1. Firstly, the top-view videos of group-housed pigs are collected. Secondly, the videos are divided into image frames. After image enhancement and segmentation, images of individual pigs are obtained. Then, the local features of pigs are extracted based on WTLD. Finally, support vector machine (SVM) classifier is used for training and recognition.

### 2.1. Experimental Setup

#### 2.1.1. Animals and Farm

**Figure 2:**
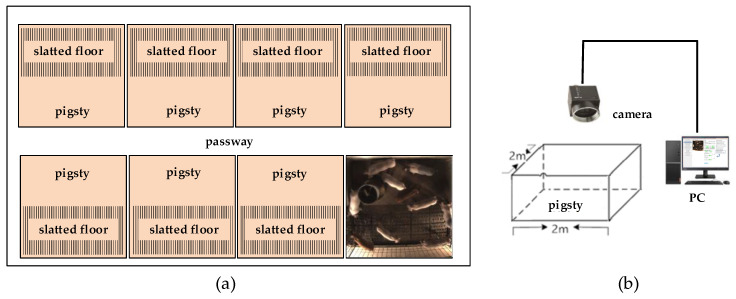
Pigsties and video capture platform. (**a**) Pigsties in the farm; (**b**) video capture platform.

Experimental videos were captured from a pig farm of the Zhenjiang Xima Development Company, based at Jiangsu University. The fattening pigsties were used in this study. There were several pigsties in the farm, which was about two meters long and two meters wide. Six to eight pigs were raised in each pigsty, as shown in Figure 2a. The breed of pigs was (Yorkshire × Landrace) × Duroc. They were 45 to 60 days old, and the average weight was about 23 kg.

#### 2.1.2. Image Collection

By rebuilding the pigsty, the camera of FL3-U3-88S2C-C from Point Grey Research Inc. (Riverside Way V6w 1k7, Richmond, BC, Canada) was installed 3 m above the experimental pigsty, which captured top-view images of group-housed pigs, as shown in Figure 2b. The resolution of the image was 1760 × 1840 pixels. Flycap2 (LUSTER LightTech group Co., Ltd, Beijing, China) of Pointgrey company was used for camera installation and configuration on the personal computer. The computer processor was the Intel^®^ Core^TM^ i7-2670QM CPU@2.2GHz (Santa Clara, CA, USA). The physical memory was 8GB and the operating system was Microsoft Windows 7. On sunny days in June 2015 and May 2017, several videos were collected from the experimental pigsty, each of which was about 3 min.

#### 2.1.3. Image Preprocessing

**Figure 3:**
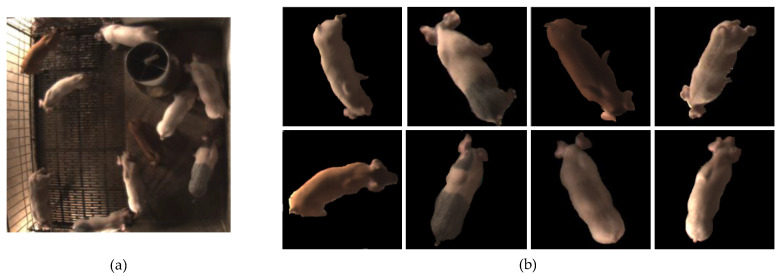
Video frame and samples of individual pig images after preprocessing. (**a**) One image frame of a video; (**b**) samples of individual pig images.

The videos were divided into image frames after collection, as shown in Figure 3a. A multi-target extraction method based on adaptive multi-threshold segmentation [24] was used to extract the image of each individual pig. Firstly, image enhancement was carried out, and the maximum entropy global threshold was used for segmentation. Secondly, the “effective region” was set, and mathematical morphology was used to obtain the initial segmentation target. According to the target centroid, the original image was adaptively divided into several circular sub-blocks. Finally, the multi-threshold local maximum was performed in the sub-blocks for the second segmentation. The images of each individual pig were normalized to the same size, as shown in Figure 3b.

#### 2.1.4. Datasets

In the experiment, two pigsties were taken as samples. In our early research, seven pigs were selected from other pigsties and mixed in pigsty 1. Their size, color, and texture on the body were more different from each other. After preprocessing, 350 individual pig images were used to establish dataset 1 for pigsty 1. Later, videos of a common pigsty named pigsty 2 was captured. There were 10 pigs bred in it, which were similar in color and body size. A total of 500 individual pig images were used to establish dataset 2 for pigsty 2. This paper takes the more general data of pigsty 2 as an example to illustrate the method; then, it applies it to pigsty 1 to solve the problem of identification of group-raised pigs in pigsty 1.

### 2.2. Weber Local Descriptor (WLD)

Psychologists have observed that the ratio of the intensity change of an object after being stimulated to its original intensity is a constant—that is, the ratio of the increasement Δ*I* to original intensity *I* is a constant *k*. This relationship is called Weber Law [25], as following:

(1) $$\frac{\Delta I}{I}=k.$$

Inspired by this, Weber local descriptor (WLD) [26] calculates the intensity difference between a central pixel and other pixels in its neighborhood. The differential excitation is used to describe the local significant pattern in the image, as shown in Equation (2):

(2) $$\xi (x_{c})=\arctan[\frac{v_{s}^{00}}{v_{s}^{01}}]=\arctan[\sum_{i=0}^{p-1}\left(\frac{x_{i}-x_{c}}{x_{c}})\right]$$

where *ξ* denotes the differential excitation, *x_c_* is the central pixel, *x_i_* is the *i*th pixel in the neighborhood of *x_c_*, and *p* represents the number of pixels in the neighborhood. $v_{s}^{00}$ and $v_{s}^{01}$ are the output of differential excitation filters *f*_00_ and *f*_01_, respectively. Then, *ξ* is evenly divided into *M* bands. Each band is uniformly quantized into *S* intervals.

In addition to the differential excitation, the gradient direction of the pixel is also calculated in WLD. The ratio of horizontal and vertical gray gradient is used to describe the local direction information in the image, as shown in Equation (3):

(3) $$\theta (x_{c})=\arctan(\frac{v_{s}^{11}}{v_{s}^{10}})$$

where *θ* denotes the direction, while $v_{s}^{11}$ and $v_{s}^{10}$ represent the output of horizontal and vertical filters *f*_10_ and *f*_11_, respectively. Then, *θ* is quantized into *T* directions after interval transformation. Finally, a two-dimensional histogram of *T* × (*M* × *S*) is constructed, where the abscissa is the direction and the ordinate is the differential excitation. Then, the two-dimensional histogram is concatenated into a one-dimensional histogram.

### 2.3. Weber Texture Local Descriptor (WTLD)

**Figure 4:**
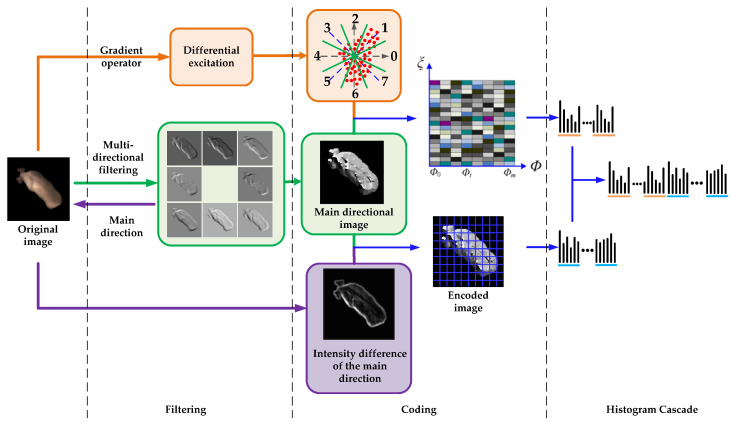
Illustration of the computation of the proposed Weber texture local descriptor (WTLD).

Although WLD computes the differential excitation and direction, only the horizontal and vertical local directions are considered, and the local structure information could not be fully expressed. In order to solve these problems, this paper proposes a Weber texture local descriptor, which not only combines multi-directional information with the differential excitation, but also contains the principal local structure information. Therefore, WTLD extracts more discriminative and powerful features than WLD. The WTLD computation is shown in Figure 4.

**Figure 5:**
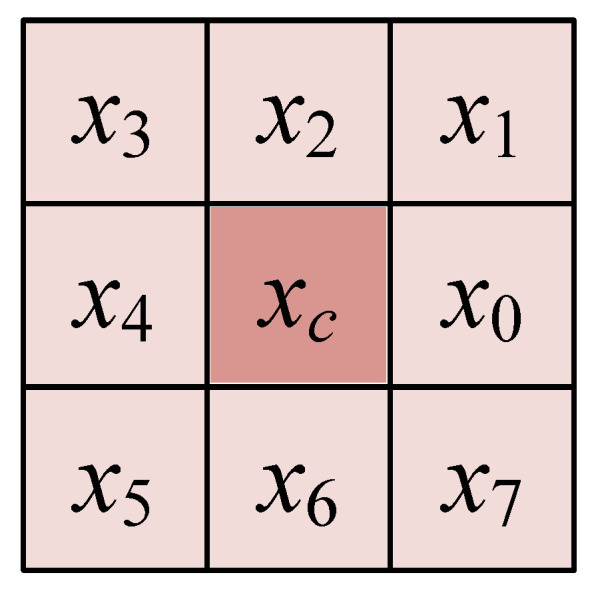
Pixel and its eight neighborhoods.

The calculation method of the proposed WTLD is as follows:

1. The differential excitation of each pixel is calculated by:

(4) $$\gamma (x_{c})=\arctan[\sum_{i=0}^{p-1}\left(\frac{x_{i}-x_{c}}{x_{c}})\right]$$

where *x_c_* represents the center pixel value, *x_i_* denotes the value of the *i*th pixel in the neighborhood, and *p* is the number of pixels in the neighborhood. Figure 5 shows pixel and its eight neighborhoods. Then, the differential excitation *γ* is evenly divided into *M* bands and each band is quantized into *S* intervals.

**Figure 6:**
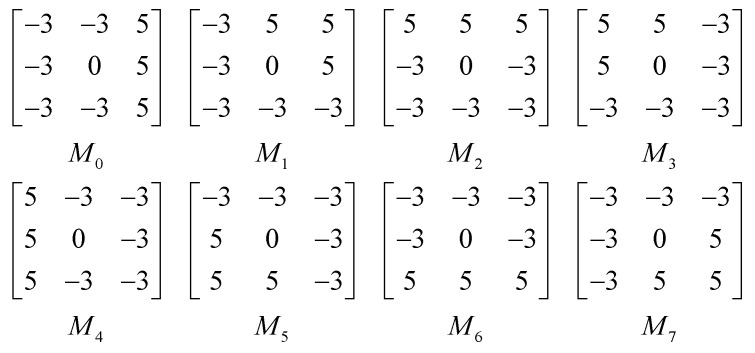
Kirsch compass masks.

2. In order to extract the local multi-directional information, the multi-directional masks are used. The original image is convoluted with the multi-directional masks, as shown in Equation (5):

(5) $$R_{i}=\left|I*M_{i}\right|,0\leq i\leq 7$$

where *I* represents the original image, *M_i_* denotes the multi-directional mask in the *i*th direction. *R_i_* is the absolute value of the filtering result in the *i*th direction. Figure 6 shows Kirsch compass masks in 8 directions.

After convoluting with multi-directional masks, the response values in multiple directions are obtained. Then, the absolute values of the directional responses are calculated. The main direction of the neighborhood, such as the maximum direction, is defined by:

(6) $$D_{1}=\;\arg\underset{i}{\max}\{R_{i}|0\leq i\leq 7\}$$

where *D*_1_ denotes the maximum directional number. In the similar way, we can obtain the second, third, and fourth maximum directional numbers: *D*_2_, *D*_3_, and *D*_4_. After that, the two-dimensional histogram of *T* × (*M* × *S*) is constructed and connected in series to form a one-dimensional histogram.

**Figure 7:**
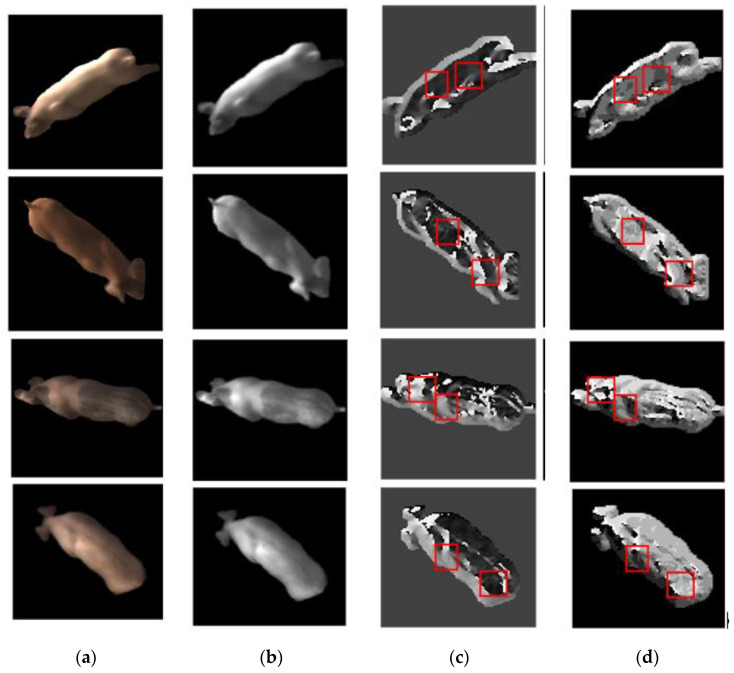
Directional images of Weber local descriptor (WLD) and the proposed WTLD. (**a**) Original images; (**b**) gray images; (**c**) directional images of WLD; (**d**) directional images of the proposed WTLD.

Figure 7 shows the directional images of WLD and the proposed WTLD. Figure 7a are original RGB images of individual pigs. Figure 7b are the gray images of the original images. Figure 7c are directional images of WLD, which are calculated by horizontal and vertical filtering. Figure 7d are directional images of WTLD. Kirsch masks are used for multi-directional filtering, and the maximum direction number is used for directional images. As can be seen from Figure 7b, the hair, skin texture, and spots on a pig’s body that are different from each other can be used for distinguishing different individuals. By comparing Figure 7c,d, it can be seen that the directional images obtained by WTLD provide more detailed local information on the pig body surface. Obvious light and shade changes can be seen in many local areas. The red squares indicate some areas, but they are not limited to these areas.

**Figure 8:**
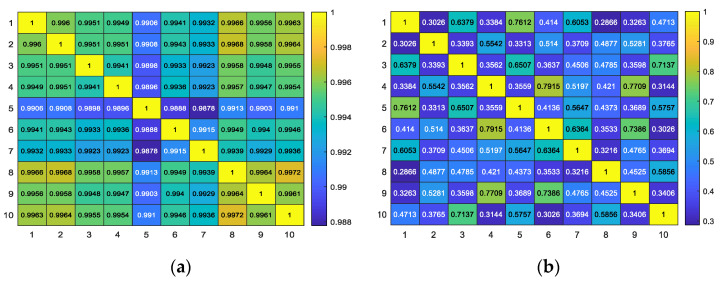
Correlation coefficient matrix of directional images based on WLD and WTLD. (**a**) Correlation coefficient matrix of directional images based on WLD; (**b**) correlation coefficient matrix of directional images based on WTLD.

In order to verify the effectiveness of the multi-directional information of the WTLD, the correlation coefficients of directional images were calculated for 10 pigs. The definition of the correlation coefficient is as follows:

(7) $$r=\frac{\sum_{m}\sum_{n}(A_{mn}-\bar{A})(E_{mn}-\bar{E})}{\sqrt{\sum_{m}\sum_{n}{\left(A_{mn}-\bar{A}\right)}^{2}\sum_{m}\sum_{n}{\left(E_{mn}-\bar{E}\right)}^{2}}}$$

where *A* and *E* are images, *m* and *n* are the size of the image, and *Ā* and *Ē* represent the mean values of *A* and *E*. Figure 8 shows the correlation coefficient matrix of directional images based on WLD and WTLD. Figure 8a is the correlation coefficient matrix of the directional images based on WLD, and Figure 8b is the correlation coefficient matrix of the directional images based on WTLD. As can be seen from the results, the correlation coefficients of different individual images based on WLD were relatively large; all the coefficients are more than 0.988. Conversely, the difference between pixels becomes larger due to the consideration of the multi-directional response of each pixel in the WTLD method. Hence, the correlation coefficient between different pig images is reduced. It indicates that multi-directional information can provide more discriminative information, which is helpful to distinguish different pig individuals.

3. The difference excitation of the original WLD only calculates the difference between the central pixel and its neighborhood. Intensity variations of pixels in the neighborhood are not considered, which resulting in an insufficient expression of local structural information. To solve this problem, the gray intensity difference between pixels in the main direction is calculated, as shown in Equation (8):

(8) $$C_{i}=\{\begin{array}{c} N_{D_{i}}-N_{D_{i}+4},D_{i}\in \{0,1,2,3\}\\ N_{D_{i}}-N_{D_{i}-4},D_{i}\in \{4,5,6,7\} \end{array},i=1,2,3,4$$

where *C_i_* is the intensity difference of pixels. The calculation of intensity difference in the main direction not only describes the maximum direction of pixel change in the neighborhood, but also distinguishes the size of the change.

Since the grayscale values can be of any size, it is necessary to quantify them for coding. Therefore, an adaptive threshold σ is adopted such that the average absolute value of the gray intensity difference in different directions are taken as the threshold, as shown in Equation (9):

(9) $$M_{i}=\{\begin{array}{ll} 1, & \left|C_{i}\right|>\sigma \\ 0, & \left|C_{i}\right|\leq \sigma \end{array},i=1,2,3,4$$

where,

$$\sigma =\frac{1}{N}\sum_{i=1}^{N-1}\left|C_{i}\right|.$$

In Equation (9), *M_i_* is the encode value of the intensity difference and *N* is 4. Then, the main direction number *D*_1_ and local structure information *M*_1_ are encoded, as shown in Equation (10):

(10) $$L(x_{c})=2\times D_{1}+M_{1}.$$

Finally, the image is divided into sub-blocks of the same size, and the local intensity histogram is calculated. The differential excitation and direction histogram are cascaded with the local intensity histogram to form a feature vector.

**Figure 9:**
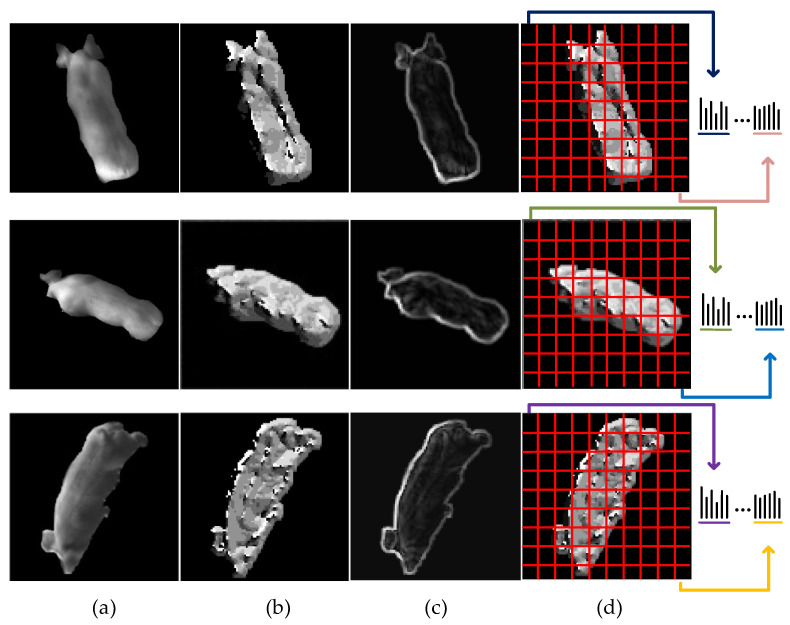
The local structure information coding process. (**a**) The gray images; (**b**) the main directional images; (**c**) the intensity difference of the main direction; (**d**) the coded images.

Figure 9 shows the local structure information coding process. As can be seen from Figure 9b,c, the main directional images reflect details such as the muscle concavity and convex, body surface patches, and so on. The intensity difference images describe more local skin texture formed by the hair. They all provide effective information to distinguish different individuals.

## 3. Experimental Results and Analysis

In this paper, two sets of data collected on the pig farm were used for the experiment. Since dataset 2 was a more general dataset in which pigs were not selected and the individual differences were not so obvious, more detailed experimental results are given with dataset 2. Then, we applied the proposed method to dataset 1 to solve the problem of identification of group-housed pigs in pigsty 1.

In the experiments, all the images were normalized to 100 × 100 pixels. Each image was divided into 4 × 4 sub-blocks to calculate the histograms. The experimental platform was MATLAB R2019b, and SVM classifier [27] with linear, polynomial and radial basis function (RBF) kernel was used for feature classification. The images were randomly divided into five groups by five-fold cross-validation. Four groups were used for training, and the remaining group was used for testing. The accuracy (Acc) on the test images was recorded and cycled five times. The average of the five results obtained on the test set was taken as the final result. Moreover, we also evaluated the performance of our model with precision (PR), specificity (SP), and F1-score (F1).

### 3.1. Comparative Experiment and Analysis of WLD and WTLD

In order to verify the effectiveness of the proposed method, Table 1, Table 2 and Table 3 show the experimental results of the original WLD, the original WLD with the local structure information added, and the proposed WTLD with different multi-directional masks. The “WLD + 1dir” and “WLD + 2dir” represent the original WLD adding the local structure information of one and two main directions, respectively. “WTLD_1dir_” and “WTLD_2dir_” represent the proposed WTLD with the local structure information of one and two main directions, respectively. The sizes of Kirsch, Sobel, and Prewitt masks are 3 × 3, 5 × 5, and 5 × 5, respectively.

It is obvious that the recognition rate of WLD is the lowest and the results of “WLD + 1dir” and “WLD + 2dir” are higher than those of WLD. It verifies that the local structural information added to WLD provides more useful information and is an effective supplement to WLD. Additionally, compared with “WLD + 1dir” and “WLD + 2dir”, the results of “WTLD_1dir_” and “WTLD_2dir_” are further improved after multi-directional filtering is adopted, which indicates that the multi-directional information extracted in WTLD also provide effective information and further enhances the expression ability of features. Furthermore, we also see that the local structure information added is not a case of the more, the better. The results of “WTLD_2dir_” are higher than “WTLD_1dir_” with Kirsch mask, while the results of “WTLD_2dir_” are lower than those of “WTLD_1dir_” with Sobel and Prewitt masks. Therefore, the local structural information of one direction is extracted and the results of WTLD means “WTLD_1dir_” in the following experiments.

### 3.2. Experimental Results of Different Multi-Directional Masks and Mask Sizes

**Figure 10:**
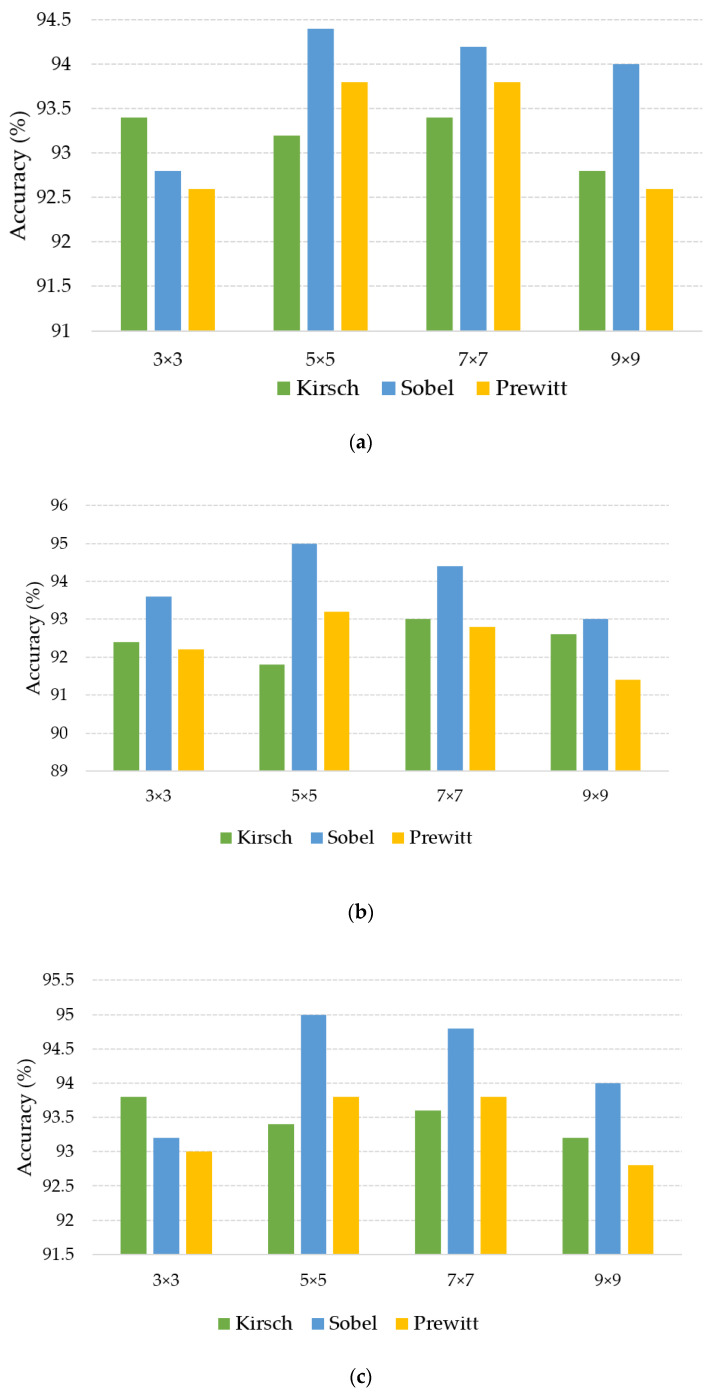
Results of different multi-directional masks and mask sizes. (**a**) Result of linear kernel SVM; (**b**) result of polynomial kernel SVM; (**c**) result of RBF kernel SVM.

In order to verify the influence of different masks and different mask sizes, multi-directional masks of Kirsch, Sobel, and Prewitt were used in the experiment. The mask sizes were 3 × 3, 5 × 5, 7 × 7, and 9 × 9, respectively. The experimental results based on linear, polynomial, and RBF kernel SVM is shown in Figure 10. As can be seen from the results, in general, the recognition rate of Sobel mask is better than those of the other masks. Sobel mask and Prewitt mask had better results when the size was 5 × 5 and 7 × 7. Kirsch mask had better results when the size was 3 × 3 with linear and RBF kernel SVM.

### 3.3. Experimental Results of Different Quantization Parameters

**Figure 11:**
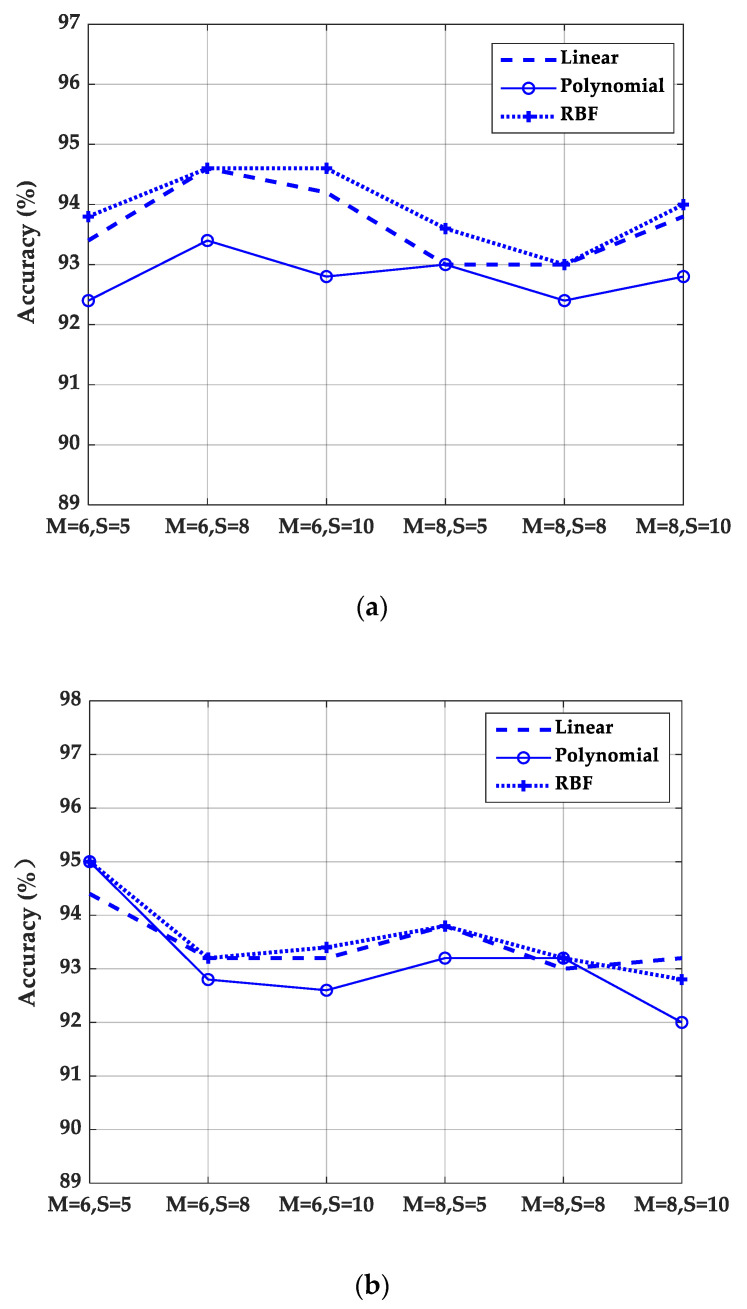

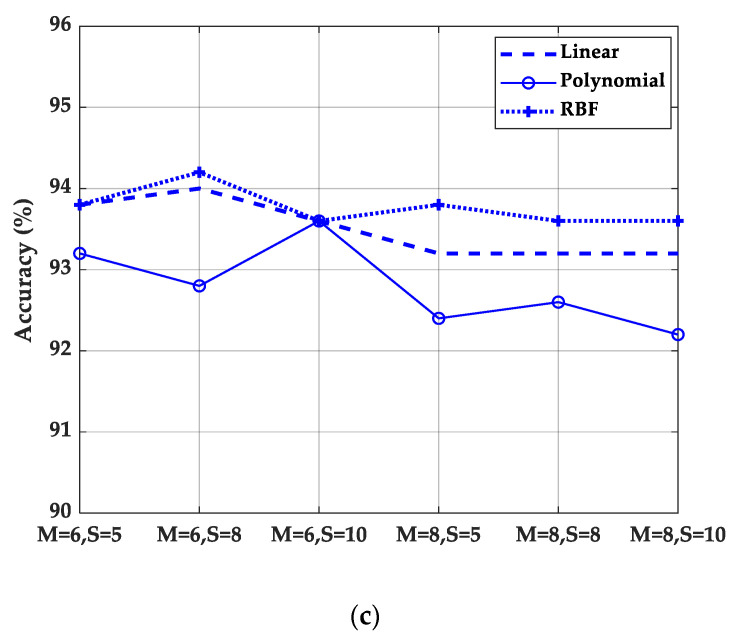
Results of different quantitative parameters. (**a**) Results of Kirsch mask; (**b**) results of Sobel mask; (**c**) results of Prewitt mask.

In order to analyze the influence of quantization parameters on the experimental results, the results of different quantization frequency bands number *M* and the uniform quantization interval number of each frequency band *S* were calculated. Figure 11 shows the results of *M* = 6, *M* = 8, and *S* = 5, *S* = 8, *S* = 10 with Kirsch, Sobel, and Prewitt masks, respectively. As can be seen from Figure 11, the results are better for Kirsch mask when *M* = 6, *S* = 8 and *M* = 6, *S* = 10. For Sobel mask, when *M* = 6, *S* = 5 the results are better, and for Prewitt mask, when *M* = 6, *S* = 8, the results are better. Overall, the results of different templates at *M* = 6 are higher than those at *M* = 8, which indicates that the quantization of differential excitation into low, intermediate, and high frequencies can effectively express the characteristics of differential excitation. The number of quantization frequency bands *M* is not a case of the more, the better. Similarly, the quantization level *S* of each frequency band is not as high as possible. With the increase of *M* and *S*, the dimension of features increases. Therefore, *M* = 6 and *S* = 5 are used in our experiments.

### 3.4. Performance Comparison Based on Different Local Descriptors

**Figure 12:**
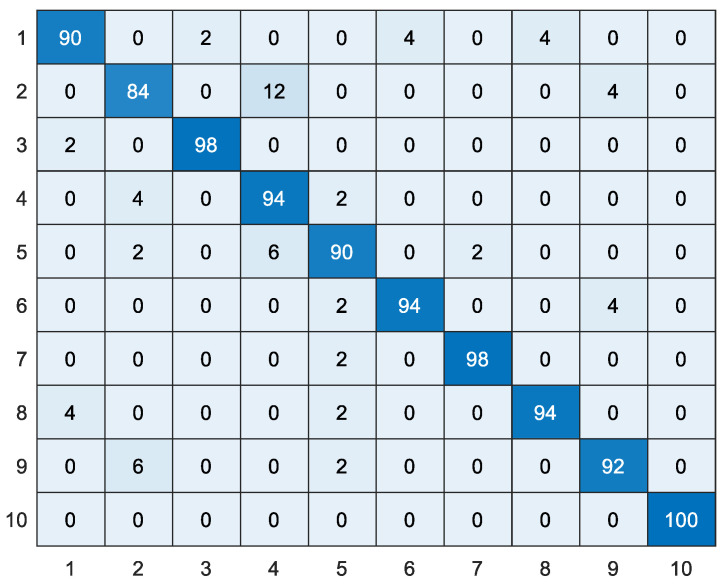
The confusion matrix obtained by WTLD_kirsch with SVM of linear kernel function (%).

**Figure 13:**
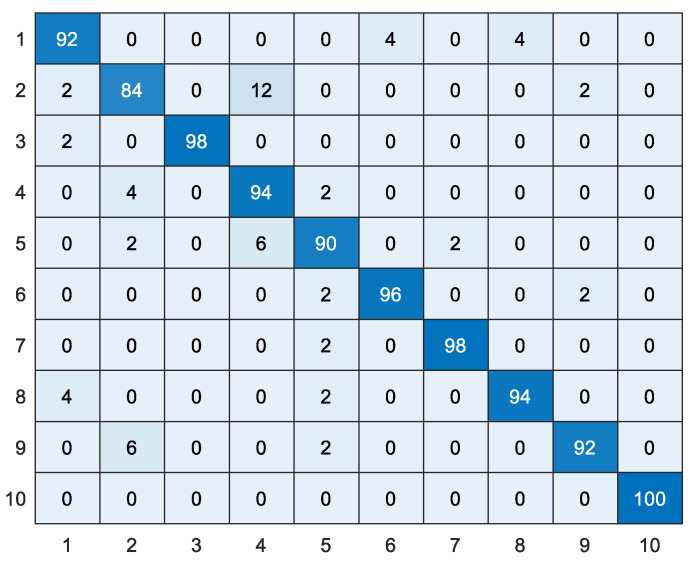
The confusion matrix obtained by WTLD_kirsch with SVM of RBF kernel function (%).

In this paper, comparative experiments were conducted between the proposed WTLD and other local descriptors of local directional number pattern (LDN) [28], local gradient increasing pattern (LGIP) [29], local binary pattern (LBP) [30], local monotonic pattern (LMP) [31], WLD [26], gradient local ternary pattern (GLTeP) [32], local arc pattern (LAP) [33], improved Weber binary coding (IWBC) [34], and median binary pattern (MBP) [35]. Table 4 shows the results of different local descriptors based on linear kernel, polynomial kernel, and RBF kernel function SVM, where the polynomial kernel is a third-order polynomial and the penalty coefficient *C* of RBF is 100. WTLD_kirsch, WTLD_sobel, and WTLD_prewitt represent the results of WTLD with Kirsch, Sobel, and Prewitt masks, respectively. The experimental results show that the proposed WTLD outperforms other local descriptors. With RBF kernel, the accuracy of WTLD_ kirsch, WTLD_ sobel, and WTLD_prewitt achieves 0.938, 0.950, and 0.938, respectively, which improves by approximately 3% compared with WLD. Additionally, it is clear that the precision, specificity, and F1-score of the proposed WTLD method are also higher than those of the other descriptors. Especially, the F1-score of WTLD_ sobel exceeds that of WLD by about 3.7%. One of the reasons is that the differential excitation and multi-directional information of pixels are calculated, and the local structure features of main direction are fused in WTLD. Therefore, a more powerful local feature descriptor is obtained. Figure 12 and Figure 13 show the WTLD confusion matrix based on linear kernel and RBF kernel SVM, respectively. The abscissa represents the actual tag, and the ordinate represents the predicted tag of the classifier.

Table 5 shows the comparison of feature dimension and feature vector length between WTLD and other local descriptors. It can be seen from Table 5 that the feature vector length of WTLD is the shortest. In WLTD, 16 × 4 × 4 is the length of the local structure information histogram, and 8 × 6 × 5 is the length of Weber’s excitation and direction histogram. Combined with the results of Table 4 and Table 5, it can be seen that the proposed WTLD has less feature dimensions and achieves higher results.

### 3.5. Results of WTLD Applied to Dataset 1

**Figure 14:**
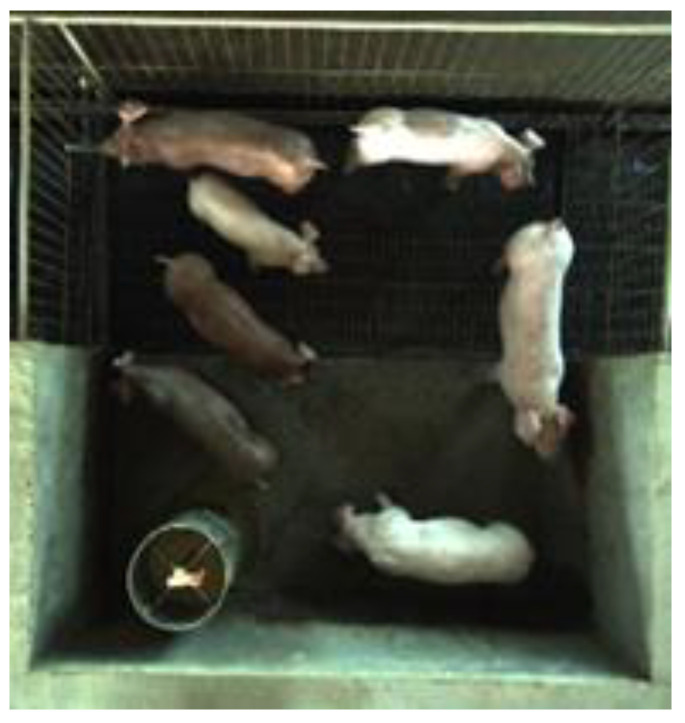
Video frame of dataset 1.

Table 6 shows the experimental results of pig identification by the proposed method in dataset 1. The experimental results also show that the proposed WTLD outperforms other local descriptors. On the whole, the experimental results of dataset 1 are higher than those in dataset 2. The accuracy of WTLD_kirsch, WTLD_sobel, and WTLD_prewitt are 97.1%, 95.71% and 97.14% with SVM of linear kernel function. The F1-scores of WTLD_kirsch, WTLD_sobel, and WTLD_prewitt with SVM of linear kernel function are 0.970, 0.954, and 0.969, which are higher than that of IWBC, which is 0.963. This is due to the obvious differences in color, texture, and spots on the body surface of the pigs in pigsty 1, which makes it easier to distinguish them from each other, as shown in Figure 14.

**Figure 15:**
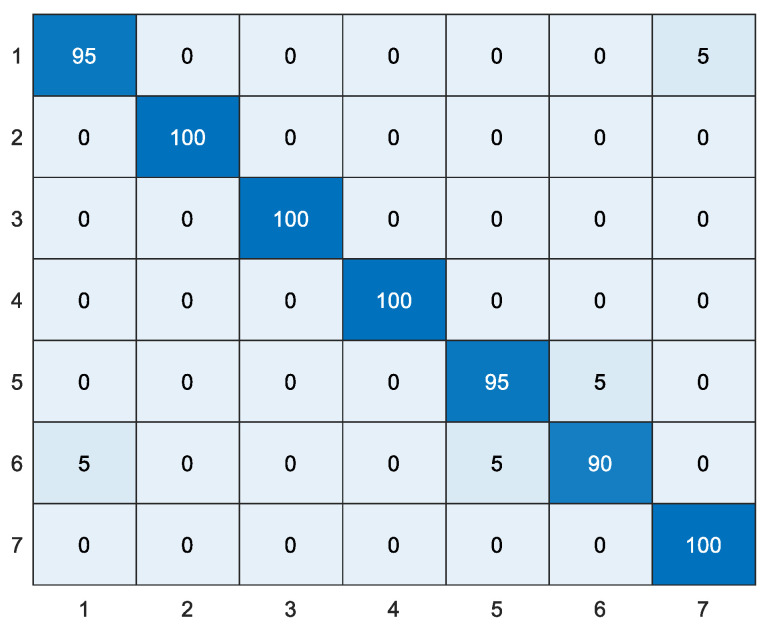
The confusion matrix obtained by WTLD_kirsch with SVM of linear kernel function of dataset 1 (%).

**Figure 16:**
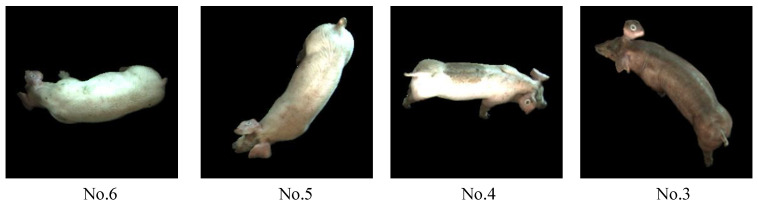
Examples of pig individuals in dataset 1.

Figure 15 shows the confusion matrix of the pig identification based on the proposed WTLD. It can be seen that pigs No. 6, No. 5, and No. 1 are easy to be misidentified as other pigs, while pig No. 2, No. 3, No. 4, and No. 7 are not easily confused. Figure 16 shows examples of pig No. 6 and No. 5. It can be seen that there is little difference in the body surface of pigs No. 6 and No. 5 from the appearance. At the same time, Figure 16 also shows examples of individuals that can easily be identified. It can be seen that pigs No. 4 and No. 3 have obvious visual features in local body pattern, skin texture, and color, so they are relatively easy to identify correctly.

## 4. Conclusions

The local features such as color change, skin texture, and spots on the body surface provide important information for the individual identification of pigs. These local features are influenced by comprehensive factors of heredity and the breeding process, which are representative. In order to realize the effective identification of group-housed pigs, a new method based on WTLD was proposed in this paper, which makes full use of the biological features of the pig body to distinguish different individuals. The multi-directional mask is applied to the calculation of Weber direction information. The local spatial domain information related to the principal direction is added. Experiments on two datasets show that the proposed method has good performance. This method establishes a model based on a pigsty that can automatically identify different pig individuals in the pigsty without requiring them to go to a specific position or maintain a specific posture. It can facilitate the identification of individual pigs with greater convenience and universality.

## Figures and Tables

**Table 1:** Comparison of experimental results between WLD and WTLD of Kirsch mask.

Method	Linear	Polynomial	RBF (*C* = 100)
WLD	91.4	90.8	91.4
WLD + 1dir	93.2	91.6	93.6
WLD + 2dir	92.6	90.4	93.2
WLTD_1dir_	93.4	92.4	93.8
WLTD_2dir_	94.0	92.4	94.0

**Table 2:** Comparison of experimental results between WLD and WTLD of Sobel mask.

Method	Linear	Polynomial	RBF (*C* = 100)
WLD	91.4	90.8	91.4
WLD + 1dir	92.8	92.0	93.0
WLD + 2dir	93.0	92.6	93.0
WLTD_1dir_	94.4	95.0	95.0
WLTD_2dir_	93.8	93.6	94.0

**Table 3:** Comparison of experimental results between WLD and WTLD of Prewitt mask.

Method	Linear	Polynomial	RBF (*C* = 100)
WLD	91.4	90.8	91.4
WLD + 1dir	93.6	91.4	93.8
WLD + 2dir	92.8	91.4	93.4
WLTD_1dir_	93.8	93.2	93.8
WLTD_2dir_	93.8	92.6	94.0

**Table 4:** Comparison of experimental results between WTLD and other local descriptors. Acc: accuracy, PR: precision, SP: specificity, F1: F1-score, LDN: local directional number pattern, LGIP: local gradient increasing pattern, LBP: local binary pattern, LMP: local monotonic pattern, WLD: Weber local descriptor, GLTeP: gradient local ternary pattern, LAP: local arc pattern, IWBC: improved Weber binary coding, MBP: median binary pattern, WTLD_kirsch: the results of WTLD with Kirsch masks, WTLD_sobel: the results of WTLD with Sobel masks, WTLD_prewitt: the results of WTLD with Prewitt masks.

Method	Linear				Polynomial				RBF (*C* = 100)			
	Acc	PR	SP	F1	Acc	PR	SP	F1	Acc	PR	SP	F1
LDN	0.890	0.897	0.988	0.889	0.862	0.868	0.985	0.857	0.886	0.895	0.987	0.885
LGIP	0.892	0.903	0.988	0.891	0.880	0.892	0.987	0.878	0.898	0.907	0.989	0.897
LBP	0.896	0.904	0.988	0.894	0.882	0.894	0.987	0.879	0.906	0.911	0.990	0.904
LMP	0.928	0.936	0.992	0.926	0.924	0.933	0.992	0.922	0.932	0.939	0.992	0.930
WLD	0.914	0.923	0.990	0.912	0.908	0.917	0.990	0.906	0.914	0.922	0.990	0.912
GLTeP	0.912	0.921	0.990	0.909	0.908	0.917	0.990	0.905	0.910	0.919	0.990	0.907
LAP	0.896	0.902	0.988	0.892	0.886	0.895	0.987	0.881	0.904	0.912	0.989	0.900
IWBC	0.930	0.937	0.992	0.928	0.930	0.938	0.992	0.928	0.934	0.941	0.993	0.932
MBP	0.894	0.904	0.988	0.892	0.882	0.891	0.987	0.880	0.898	0.908	0.989	0.896
WTLD_kirsch	0.934	0.941	0.993	0.933	0.924	0.933	0.992	0.922	0.938	0.944	0.993	0.937
WTLD_sobel	0.944	0.949	0.994	0.943	0.950	0.957	0.994	0.950	0.950	0.955	0.994	0.949
WTLD_prewitt	0.938	0.945	0.993	0.938	0.932	0.939	0.992	0.931	0.938	0.944	0.993	0.937

**Table 5:** Comparison of feature dimension and feature vector length between WTLD and other local descriptors.

Method	Feature Dimension	Eigenvector Length
LDN [28]	56	896
LGIP [29]	37	592
LBP [30]	59	944
LMP [31]	256	4096
WLD [26]	32	512
GLTeP [32]	512	8192
LAP [33]	272	4352
IWBC [34]	2048	32,768
MBP [35]	256	4096
WTLD	256 (16 + 8 × 6 × 5)	496 (16 × 4 × 4 + 8 × 6 × 5)

**Table 6:** Comparison of experimental results between WTLD and other local descriptors of dataset 1.

Method	Linear				Polynomial				RBF (*C* = 100)			
	Acc	PR	SP	F1	Acc	PR	SP	F1	Acc	PR	SP	F1
LDN	0.914	0.926	0.986	0.911	0.921	0.936	0.987	0.919	0.921	0.928	0.987	0.917
LGIP	0.914	0.933	0.986	0.909	0.907	0.927	0.985	0.902	0.914	0.933	0.986	0.909
LBP	0.900	0.908	0.983	0.896	0.900	0.908	0.983	0.896	0.900	0.908	0.983	0.896
LMP	0.950	0.964	0.992	0.948	0.950	0.964	0.992	0.948	0.950	0.964	0.992	0.948
WLD	0.921	0.940	0.987	0.916	0.914	0.934	0.986	0.909	0.921	0.940	0.987	0.916
GLTeP	0.950	0.958	0.992	0.948	0.950	0.958	0.992	0.948	0.950	0.958	0.992	0.948
LAP	0.893	0.900	0.982	0.888	0.893	0.903	0.982	0.888	0.893	0.903	0.982	0.888
IWBC	0.964	0.973	0.994	0.963	0.964	0.973	0.994	0.963	0.964	0.973	0.994	0.963
MBP	0.929	0.933	0.988	0.927	0.929	0.933	0.988	0.927	0.929	0.933	0.988	0.927
WTLD_kirsch	0.971	0.979	0.995	0.970	0.971	0.979	0.995	0.970	0.971	0.979	0.995	0.970
WTLD_sobel	0.957	0.968	0.993	0.954	0.957	0.968	0.993	0.954	0.964	0.973	0.994	0.963
WTLD_prewitt	0.971	0.979	0.995	0.969	0.971	0.979	0.995	0.969	0.971	0.979	0.995	0.969
